# Complexity on the Menu and in the Meal

**DOI:** 10.3390/foods7100158

**Published:** 2018-09-27

**Authors:** Charles Spence

**Affiliations:** Department of Experimental Psychology, New Radcliffe House, University of Oxford, Oxford OX2 6BW, UK; charles.spence@psy.ox.ac.uk; Tel.: +44-1865-271364

**Keywords:** complexity, mixture perception, recipe, menu design

## Abstract

Complexity is generally perceived to be a desirable attribute as far as the design/delivery of food and beverage experiences is concerned. However, that said, there are many different kinds of complexity, or at least people use the term when talking about quite different things, and not all of them are relevant to the design of food and drink experiences nor are they all necessarily perceptible within the tasting experience (either in the moment or over time). Consequently, the consumer often needs to infer the complexity of a tasting experience that is unlikely to be perceptible (in its entirety) in the moment. This paper outlines a number of different routes by which the chef, mixologist, and/or blender can both design and signal the complexity in the tasting experience.

## 1. Introduction

Complexity is commonly talked about as a desirable attribute of the consumer’s experience of food and drink (e.g., Kramer, 2012, [1], Passmore, 2015, [2]). In fact, if we go back more than half a century, we find Singleton and Ough (1962, p. 189) suggesting that: *“Complexity has long been considered a desirable factor in the quality of most flavorsome or odorous products.”* [3]. Marketers (e.g., in the world of wine) believe that consumers are willing to pay for complexity (Parr, 2015, [4]). It is, after all, one of the ways that demonstrates the skill of the chef, mixologist, or blender (cf. King, 2014, [5]). Complexity (at least in the world of wine) is also an attribute that is linked in many people’s minds to the notion of quality (e.g., see Spence & Wang, 2018, in press; [6,7]) Just take the following: “*The single greatest standard used in assessing the quality of a wine is complexity. The more times you can return to a glass of wine and find something different in it—in the bouquet, in the taste—the more complex the wine. The very greatest wines are not so much overpowering as they are seemingly limitless.”* [2]. That said, it is not always altogether clear the extent to which different people are necessarily using the term ‘complexity’ to refer to the same thing (see Parr, 2015, Spence & Wang, 2018, [4,6,7]).

Furthermore, complexity is often not perceptible subjectively. That is, it is often not perceptible sensorially to the diner/drinker in their tasting experience, rather it is inferred (Spence & Wang, in press) [7]). What, then, is the chef, mixologist, blender, culinary creative, or marketer to do in order to ensure that those who are tasting their food appreciate or, at the very least, are aware of, the full complexity that may be present in the making of the meal or in the mixing/blending of the ingredients in a dish or drink? In this review, a number of the different ways in which the term complexity is used when talking about food and drink experiences are highlighted. I also review some of the different ways in which those working in food and beverage design go about signaling the complexity that so often lies behind their (sometimes imperceptible) craft.

## 2. Complexity in the Making/Preparation

Prior to the diner/drinker tasting the food or beverage product, there are a number of ways in which those preparing/creating it can signal the complexity of the process that leads to its creation (cf. Biderman, 2017) [8], according to the introduction to Ulijaszek, Mann, and Elton’s (2012, p. 12) volume *Evolving Human Nutrition*: *“Without a large neocortex, humans could not have elaborated cuisines, not only because cooking can be a complex process”* [9]. Below, I start by reviewing the way in which complexity is highlighted on the menu before looking at the complexity that may be present in the preparation of a dish or drink.

### 2.1. Complexity on the Menu

The presence of complexity on the menu can, of course, be signaled simply by listing out the multitude of ingredients/elements that went into making a dish. That said, oftentimes, it feels more like the aim is to highlight some of the more/most expensive/luxury elements in the dish rather than to overwhelm the diner with a long list of ingredients. Research from Dan Jurafsky (2014) has shown that the more letters on the restaurant menu, the more a meal is likely to cost [10]. In particular, according to one analysis of online restaurant menu descriptions, the average price of a dish in the US was found to go up by 6 cents for each additional letter in the dish description. Given such results, one might well think it sensible for those designing restaurant menus to go for ever more elaborate descriptions on their menus as a means of increasing their bottom line. Along these lines, preliminary research from Mielby and Bom Frøst (2012) [11] has suggested that diners like it when complex modernist cooking techniques are described on the menu (or, at least, that was the state of affairs a few years ago).

McCall and Lynn (2008) conducted an intriguing study to assess the impact of menu complexity on people’s perception of quality, price, and purchase intention in a relatively large sample of 160 college students [12]. Responses were made on nine-point rating scales. Away from the restaurant environment, the participants were shown restaurant menu descriptions for three main course items—*Filet Mignon*, *Stuffed Breast of Chicken*, and *Pasta*. The items had either simple or complex descriptions (see Table 1). The results clearly showed that the dishes given more complex menu descriptions were rated as more desirable in terms of their perceived quality. Participants also expected to pay more for the dishes and they reported a higher purchase intent as well. Such results would, therefore, appear to support the suggestion of many restaurant consultants that complex wording should be used on the menu in order to communicate the distinctiveness or unique character of the cuisine.

That said, one has to be careful since an overly descriptive label can all too easily come across as pretentious (see Spence & Piqueras-Fiszman, 2014) [13]. For instance, just take the following imaginary dish from the British restaurant critic (for *The Times* newspaper) Giles Coren: “*Lobster Pierre Choderlos de Laclos, sauce Antoine de Saint-Exupéry, sur son lit de pommes de terre façon, merde, je ne me rappelled plus le nom, tu sais, le mec qui etait le chef de Tallyrand, je voulais dire Antonin Worrall-Thompson, mais ce n’est pas lui*” (Coren 2012, p. 176) [14]. That is very much the point here.) The identity of the dish looks incomprehensible and complex and that is very much the idea, no matter whether one understands French or not. In recent years, the brief description of a dish: “Balls”, ‘Fish” … has also become increasingly fashionable (see Halligan 1990, p. 198, Spence & Piqueras-Fiszman, 2014) [13,15]. Ironically, however, the very absence of adjectives in a dish’s description can itself become problematic as Giles Coren (2012, p. 177) has pointed out: “*Brevity and the low vernacular: ‘beef in crust,’ ‘burnt cream,’ ‘cock in wine.’ It’s a weird, strangulated form of pretentious unpretentiousness. And it feels to me like the most pretentious sort of naming there could be*.” [14]. The growing popularity of shorter dish names may, in other words, mean that it is currently not as simple as it once was to signal complexity through the extended wording of the menu item.

### 2.2. Complexity in the Making/Process

The rise of the open kitchen has undoubtedly facilitated the diner’s ability to see the culinary artistry (and complexity therein). That said, the cooking process normally takes place at too slow a pace for the diner to really get a proper sense of all the steps that may be involved in the preparation of a dish. Hence, the emergence of the open kitchen is perhaps better framed as a means of signaling that the food that eventually appears on the dining table has been freshly prepared (and playing to the growing interest in food theatre) than anything else (see Spence, 2017) [16]. Note here that complexity can also be present in/signaled by the service encounter. However, unfortunately, this is an area where I have been unable to find any relevant research. One might also think of the signaling regarding the complexity in terms of the recipe descriptions. Just think about all those cookbooks packed full of glorious ‘food porn’ and recipes that one wonders how many diners ever actually cook (e.g., see Levy, 2013) [17]. At least 24 h worth of preparation is what some commentators have estimated that it would take them if they were to follow certain types of recipes precisely. According to Levy’s (2013) description: *“Among the recipes it contains is one for a wonderful dish served at Dinner: “Meat fruit”, a life-like tangerine with an aromatic orange zest, topped by a stem with a couple of leaves.”* The journalist continues *“The instructions for making it run to more than 1000 words, take a few days (“allow the mandarin jelly to stand in the fridge for a minimum of 24 h before using”) and require a sous-vide sealing machine that removes the air from plastic bags, a temperature-controlled water bath, and a Thermomix that blends and heats simultaneously. Plus heat probes, silicon moulds–oh, and scales can weigh out as little as 2 g. The book itself is beautifully designed, bound, slip-cased, and illustrated. As must be evident, you can’t really use it to cook from at home. Not only is the domestic batterie de cuisine not up to the job; who has the time for such labor-intensive preparations?”* [17]. In this scenario, the point of all that complexity would seem to be merely to signal the skill of the chefs in the kitchen and, thus, their real purpose may well be to reinforce the value (and presumed complexity) of the meals served in their restaurants as much as anything else. Hence, even if the diner cannot see all that complexity at work in the open kitchen, as long as they have skimmed the star chef’s latest cookbook, they may implicitly know what a laborious set of procedures and techniques are likely at work behind the scenes in the restaurant.

### 2.3. Complexity in the Meal/Dish

At this point, one might consider chef Ben Spalding’s dish at the short-lasting John Salt restaurant in Islington, North London (http://john-salt.com, Masters, 2012, O’Loughlin, 2012) [18,19]. In this case, the chef made a salad consisting of a wide variety of different leaves/herbs. The dish was memorably served to diners accompanied by a London Tube Map on which the various underground stations had been replaced by the names of the leaves/herbs that might, depending of the season/availability, be found in the salad. The dish is undoubtedly complex at least in terms of the number of distinct elements involved, but the unusual use of the Tube Map also brought a note of playfulness/levity to the dish, which avoids the pretentiousness that might have been associated with the dish had the chef simply listed out all of the leaves in his salad. 

Alternatively, here, we also have chef Andoni’s 100-element salad served at his restaurant Mugaritz in San Sebastian, Spain (see Aduriz, 2014) [20]. In this case, the salad is even more complex (at least if complexity is judged by the number of elements in the dish), but it would seem more like a random combination of seasonal leaves/herbs/flowers than necessarily a carefully constructed flavor experience. This is a point emphasized by the fact that the elements are simply tossed together seemingly without care for their precise arrangement. One might, therefore, want to ask what the purpose of putting so many elements into a dish is, if the diner cannot necessarily identify them? One can also think about the complexity of the organization of the various elements on the plate too (cf. Zellner, Lankford, Ambrose, & Locher, 2010) [21]. The plating of some of the most beautiful dishes prepared by French chef Michel Bras (http://www.bras.fr) are worth mentioning here. He uses what he calls ‘negative space’ (playing with the contrasting background elements) in order to accentuate the many colorful ingredients that he works with. In addition, although these elements may seem to have been placed spontaneously on the plate, they actually require around 100 individual actions. What may look random/accidental is, in other words, actually a very complex and highly-orchestrated procedure. 

The plating and visual presentation of restaurant dishes has become increasingly complex in recent years (see Deroy, Michel, Piqueras-Fiszman, & Spence, 2014, Spence, Piqueras-Fiszman, Michel, & Deroy, 2014, for reviews) [22,23]. A couple of the latest dishes from chef Jozef Youssef of Kitchen Theory play with visual illusions on the plate using the Gestalt principles of emergence (Spence & Youssef, 2016), and bi-stability (Youssef, Sanchez, Woods, & Spence, 2018). These dishes are conceptually complex, but this complexity occurs independently of the complexity of the ingredients/flavors/tasting experience involved [24,25].

### 2.4. Complexity in Terms of the Structure of the Meal

As chefs have increasingly gone from the traditional three, five, or seven course meal up to tasting menus with 20 or more dishes, one is reminded here of El Bulli’s closing menu with 49 courses (Edwards, 2011, see also Aron, 2015 or Bompas & Parr’s one-off 200 course meal served over a 24-h period) [26,27]. It should, though, perhaps be noted that several of the ‘courses’ actually consisted of cocktails. Here, the complexity is, in part, revealed by the multitude of dishes/flavors that assault the diner’s palate over the course a multi-hour dining experience. Many Western chefs have enthusiastically embraced this Japanese tradition (see Booth, 2010) [28]. However, as a growing number of chefs have come to realize, they need to add structure to the meal/menu in order to prevent it from becoming an unmemorable (or worse still, potentially bewildering) list of the chef’s greatest hits. That said, one of the problem here is how to avoid satiety setting in before the proceedings have drawn to a close (see Piqueras-Fiszman & Spence, 2014, Ruijschop, Boelrijk, Burgering, de Graaf, & Westerterp-Plantenga, 2010) [29,30]. Here, it is perhaps worth noting that prior to the switch from service *à la Russe* (i.e., Russian-style) to service *à la Française* (or French-style, that is, from the simultaneous to the sequential presentation of the dishes) court banquets might consist of many tens of courses too (see Visser, 2009) [31], which are carefully arranged on the table at the same time but without any obvious structure/organization to the sequences of flavors that the diner might experience.

### 2.5. Complexity in the Mixing

Switching for a moment from dining to drinking, there is relevant discussion here around the world of mixing and blending. For instance, Johnnie Walker (Blue label) whisky is made of a blend of 35 different single malt whiskies. One might compare this to the many botanicals that may be advertised in a premium gin and wonder which spirit is more complex (Spence, in press) [32]. In the case of blended whiskies and wines, it can be incredibly complex to make an end product that tastes the same as it always has (i.e., in years gone by) despite the fact that the raw materials may change from one year to the next (see Smith, Sester, Ballester, & Deroy, 2017, on blended whiskies, cf. Harrar, Smith, Deroy, & Spence, 2013, on blending in Champagne) [33,34]. The real challenge here is the very unpredictability of what happens when different olfactory stimuli are mixed together (e.g., see Dubow & Childs, 1998, Thomas-Danguin, Barba, Salles, & Guichard, 2017) [35,36].

The notion that the art of blending involves the obscuring of some of the individual elements links to what is often said about a good Indian curry. While the latter may well contain 20–30 or more herbs and spices and so is likely more chemically complex than many other dishes, it is commonly held that the chef has failed if it is possible to taste the individual elements (see Adapon, 2008, Jain, Rakhi, & Baglerb, 2015) [37,38]. One has to be a little careful in terms of one’s terminology here given that the term ‘curry’ is a Western catch-all for a range of dishes/styles of cuisine (see Howes & Classen, 2014, p. 87) [39]. According to Wright (2010, p. 6): *“If a natural* (flavor) *character is desired, then the optimum level of complexity is often the minimum number of components required to prevent the taster from perceiving the individual characters. This level of complexity can vary from perhaps as few as 15 components in a simple fruit flavor to up to 100 in the most complex flavor of cooked food.”* [40]. Nevertheless, this bringing together and combining of multiple separate elements in a dish would seem to be one of the ways in which people commonly use the term ‘complexity.’ Singleton and Ough (1962, p. 196) noted more than half a century ago when describing the increased ratings of quality seen when they blended pairs of single varietal wines together: *“A further implication is that a flavor that may be undesirable when recognizably strong may be a contributor to complexity and, therefore, not undesirable if below the recognition threshold in the blend.”* [3].

## 3. Complexity in the Meal

Complexity in the meal itself (as seen from the diner’s perspective) can occur at various levels from the granular to the high-level.

### 3.1. Molecular Complexity

The chemists have various formulae by which to assess the complexity of a molecule (e.g., see Hendrickson, Huang, & Toczko, 1987, for one example) [41]. What is more, those molecules that are more complex tend to, on average, give rise to a greater number of odor descriptors (Kermen, Chakirian, Sezille, Joussain, Le Goff, Ziessel, Chastrette, Mandairon, Didier, Rouby, & Bensafi, 2011, cf. Khan, Luk, Flinker, Aggarwal, Lapid, Haddad, & Sobel, 2007) [42,43]. That said, it should also be remembered that many simple molecules can also give rise to complex percepts. They have also been shown to give rise to more activity in brain areas such as the anterior cingulate (a part of the brain that is normally involved in conflict resolution. Sezille, Ferdenzi, Chakirian, Fournel, Thevenet, Gerber, Hummel, & Bensafi, 2014) [44]. However, this is of less relevance here given that no food or beverage products are constituted entirely of just a single molecule. Even water, which is often sold as ‘pure,’ isn’t actually pure H_2_O. If it were, it would taste bitter/sour (Engber, 2014, Lewis, 2017, Zocchi, Wennemuth & Oka, 2017) [45,46,47]. Instead, various different minerals are added to match the composition of saliva and deliver a more-or-less neutral taste (Logue, 2004, Spence, 2011) [48,49]. Hence, while objectively measurable, this kind of chemical (i.e., molecular) complexity is only really of theoretical interest to us here.

### 3.2. Complexity in the Mixture

Most food and drink products actually contain many tens if not hundreds of different molecules (e.g., Bensi, 2008, Maarse, 1983) [50,51]. A quality wine or a specialty coffee, for instance, may well contain anywhere between 600 to 1200 volatile molecules (e.g., Clarke, 2013; Rapp, 1990; Tao & Li, 2009) [52,53,54]. A number of commentators have wanted to link the notion of complexity with the number of volatile organic compounds that one finds in a food product e.g., Gill, 2008, Kew, Goodall, Clarke, & Uhrín, 2017, Thorngate, 1997) [55,56,57]. That said, it is important to note that only 30 to 40 of those molecules will likely influence the perceived aroma/flavor (Bensi, 2008) [50]. Flavor chromatography is a popular technique to identify the volatiles in a food. This chemical analysis technique is normally combined with a sensory correlation technique in order to provide information about what the average consumer perceives. In addition, as we will see below, cognitive limitations on the decoding/perception/interpretation of multi-odor mixtures can be even more limiting.

### 3.3. Complexity in the Moment

Reading the wine press, one could all too easily be convinced that there is a world of complexity in a quality glass of wine. For example, Smith (2008) highlights the following beautiful example from Robert Parker’s website (eRobertParker.com): *“I had to draw a deep breath in anticipation of what would rise from my glass of Leroy 2005 Musigny and, in that moment, Madame Leroy trenchantly observed that ‘it’s another world’ down in there. Tea, ginger, tangerine rind, myriad sweet flowers, cherry distillate, truffles, and fraise des bois preserves are among the scents that pour forth. Bloody fresh meat and implacable chalk join the medley of fruit, floral, and spice concentrates on a palate whose texture “polish,” “refinement,” or “velvet” are pitifully inadequate to describe. For all of its amazing richness, this displays more of a confident inner sweetness than a superficial sucrosity and there is no lack of sheer energetic brightness of fruit and drive. Indeed, seatbelts would be advised before attempting to swallow this elixir such is the phenomenal thrust of its finish.”* [58]. However, by contrast, well-controlled laboratory research suggests instead that three is about the limit in terms of the number of discrete notes/or elements that the taster (even expert taster) can pick out of a mixture of as many as six odorants and/or tastants (at least from a single sniff) even if the latter happens to be very familiar with all of the odorants when presented individually (Ferreira, 2012a, b, Frank, Fletcher, & Hettinger, 2017, Jinks & Laing, 1999, 2001, Laing, Link, Jinks, & Hutchinson, 2002, Marshall, Laing, Jinks, & Hutchinson, 2006) [59,60,61,62,63,64,65].

This discrepancy might be explained in a number of ways. It might, for instance, simply be that real foods vary in many other sensory attributes such as, for instance, their oral-somatosensory texture/mouth feel (Gawel, Oberholster, & Francis, 2000, see Spence & Piqueras-Fiszman, 2016, for a review) [66,67] not to mention the color etc. (Fielden, 2009, Spence. 2010a, b) [68,69,70]. What is more, it is often suggested in the case of wine (or at least in the case of a quality wine) that it evolves on the palate and in the glass. It evolves in the case that the bottle once opened / in the decanter and over the lifetime of the product as it ages in the cellar/in the barrel (e.g., Boulton, Singleton, Bisson, & Kunkee, 1999, Ribéreau-Gayon, Glories, Maujean, & Dubourdieu, 2006, Spence, 2011) [71,72,73] Here, it is worth noting that complexity may not only emerge from the sequential sampling of the wine as it ages but also be a perceptual quality that emerges as a result of aging. Take Singleton and Ough (1962, p. 189) who noted more than half a century ago that: *“In wines, flavor complexity is considered very important to high quality and is believed to be one of the primary effects produced by proper aging.”* [3].

One might also think of literature suggesting that two palates (or at least minds, in the case of perceptual decision making. Bahrami, Olsen, Latham, Roepstorff, Rees, & Frith, 2010, Brennan & Enns, 2015, Koriat, 2012, 2015) [74,75,76,77] may be better than one and that perceived attributes of the wine might be imagined (cf. Berger & Ehrsson, 2013, Spence & Deroy, 2013) [78,79] filled-in (cf. Pessoa & De Weerd, 2003; Väljamäe & Soto-Faraco, 2008) [80,81] or else completed in a way that is perceptually real to the taster (see Spence & Wang, in press). The expert wine taster will, for instance, associate clusters of features so that, if they smell cassis and know that they are drinking a cabernet sauvignon, then they may well also rattle off notes of green pepper and mint and leather, etc. Whether they actually perceive these notes or whether this is instead a matter of mental imagery, perceptual completion, or filling-in is, I would like to argue, an open question (Spence & Wang, in press) [7]. Hence, one could also imagine a taster working with someone else’s tasting notes that they respected (or even with their own notes from an earlier point in time. Herzog & Hertwig, 2009) in order to obtain a much richer impression of what they believe that they can smell in the glass or at least what they expect to be present in a wine [82]. Thereafter, the taster’s imagination may well do the rest in terms of filling-in the complex array of aroma notes that they expect to be present in the wine (see Spence & Wang, in press) [7]. Of course, one also needs to consider the possible influence of bias here.

The notion of chunking from the field of cognitive psychology may also be relevant here too (e.g., Gobet, Lloyd-Kelly, & Lane, 2016) [83]. Additionally, this is presumably also part of what is lost (i.e., what laboratory experiments fail to capture) when odorants are mixed randomly in the laboratory. By analogy, chess experts, for instance, can chunk together and, hence, remember the positions of many more pieces from a real game board than amateurs. By contrast, they are no better than amateur chess-players in remembering the positions of the pieces if they have been placed randomly on the board (Chase & Simon, 1973) [84].

Another way in which to think about subjectively-perceived flavor complexity is in terms of configural processing (Jinks & Laing, 2001, Parr, 2015) [4,63]. Studies of wine tasters among both social drinkers and wine-experts have revealed that, while they sometimes want to ascribe complexity to a tasting experience where there is a lot going on, at other times, they ascribe the descriptor complexity to a unitary flavor experience (Parr, Mouret, Blackmore, Pelquest-Hunt, & Urdapilleta, 2011, Schlich, Maraboli, Urbano, & Parr, 2015) [85,86]. Parr (2015) has the following to say: *“In other words, the integration of aromas in a multi-component mixture such as a wine … may be considered a higher level of abstraction that can give rise to a single perception described by the word “complex.” This higher level of abstraction is argued as involving configural perception or perceptual fusion where the multiple components of a stimulus are recognized as a whole pattern with the individual components not necessarily accessible to consciousness. That is, as distinct from elemental perception where the components of a mixture such as a wine are perceived within the mixture, configural perception involves blending of the components or properties of the mixture with the resulting percept or Gestalt being different from the components.”* [4]. There is an important distinction here between complexity judgments ascribed to a particular unitary flavor experience or in the configuration of parts. Indeed, Spence and Wang (in press) have recently drawn attention to a potential distinction between inferred complexity and complexity that is directly perceived [7].

However, while people may want to ascribe complexity to a flavor experience with only a single dominant note, it is certainly not clear that the configural approach is necessarily the best way to think about what may be going on. This is because, in vision, the configural account is much more highly developed (e.g., as in the study of face perception) while the elements may be grouped as a configural whole. The individual elements retain their identity in a way that is not so obviously the case in flavor perception (see also Kay, Crk, & Thorngate, 2005, Romagny, Coureau, & Thomas-Danguin, 2017) [87,88]. One might extend the configural account to think of a harmonious grouping of flavors and/or a configuration of distinct flavor objects (see Spence, Wang, & Youssef, 2017, on the topic of flavor pairing where this kind of account also crops up) [89]. Whatever the explanation, it is clear that there are both profound cognitive/physiological limits on the ability to perceive complexity in the moment and different things being referred to by those who use the term (see also Veinand, Deschamps, Mandon, & Adam, 2018) [90].

Complex experiences are unlikely to be processed fluently while simple dishes/flavors by contrast are processed fluently (e.g., Reber, Winkielman, & Schwartz, 1998) [91]. One of the ways in which chefs disrupt processing fluency and, hence, potentially deliver a more complex tasting experience, is by using sensory incongruency in a dish. A number of molecular/modernist chefs often play with foods that look like one thing while tasting like another (see Piqueras-Fiszman & Spence, 2012, Velasco et al., 2016, for reviews) [92,93]. Such experiences are complex in that the diner needs to figure out the intentions behind the dish in order to fully enjoy it as a designed experience and not simply think of it as a poorly designed or executed dish. As Michelin-starred Spanish chef Andoni Luis Aduriz put it a few years ago: *“You know, I went to cooking school decades ago and there they taught me how to make delicious food. It’s not my goal to make delicious food anymore. I want to make interesting food.”* (quoted in Ulla, 2012) [94].

### 3.4. Complexity in the Mouthful

Chef Denis Martin’s serves a *Thon au chocolat blanc et piment Thaï* dish at his namesake restaurant in Vevey, Switzerland (http://www.denismartin.ch/; Martin, 2007) [95]. The dish is consumed in a single mouthful, which delivers a distinctive series of flavor experiences that extend over time. First comes the sharp ‘hit’ of wasabi, then the tender texture of the tuna, and, finally, the slow creamy melting mouthfeel of the white chocolate. The various flavors/textures/mouth feels evolve over the course of a dish that consists of a single mouthful of food (see Spence et al., 2017) [89]. It has been argued elsewhere that it is really the temporal evolution of different sensory elements in the tasting experience where much of the perceived, or inferred, complexity is revealed (Kramer, 2012, Smith, 2014, Spence & Wang, 2018) [1,6,96].

## 4. Conclusions

While consumers undoubtedly do value complexity as far as their experience of food and drink are concerned (Passmore, 2015) [2], that complexity is typically not subjectively perceptible in the mind of the taster in the moment. Consequently, there exist a number of different routes by which the creator/marketer of a dish/drink may be able to signal the complexity that was involved in the art of making/blending. Ultimately, given the limitations of our perceptual abilities/information processing capacities as far as the chemical senses are concerned (see Gallace Ngo, Sulaitis, & Spence, 2012, for a summary [97], see also McGann, 2017) [98], perceived complexity is normally likely to be something that emerges over time rather than being perceptible in the moment (e.g., Smith, 2014, Spence & Wang, 2018) [6,96]. That said, the timeframe of that temporal evolution of multi-element complex tasting experiences may take place over the course of a prolonged mouthful (Martin, 2007, Spence et al., 2017) [89,95] over the course of a dish or drink, over the course of an entire meal, and/or over the lifetime of the product itself (e.g., in the case of a quality wine as it ages, Kramer, 2012) [1].

We value subjective complexity but that is something that is often not perceptible in the mix. No wonder then that stress is placed on complexity on the making, be it in terms of the number of ingredients on the menu, the number of steps in the recipes in the cookbooks that perhaps no one ever cooks from, the number of courses, or the skill of the maker to have their work imperceptible. While complexity has primarily been framed as positive here, one might, in closing, want to question whether it should always be thought of as such. Here, it is just worth noting the striving for simplicity that is undoubtedly a key feature that many chefs are striving toward these days. Early work on complexity in the visual arts suggested an inverted U-shaped graph linking liking with complexity (Berlyne, 1960; Berlyne, Ogilve, & Parham, 1968; Wang & Spence, 2018) [99,100,101]. As such, one might imagine that there are certain flavor experiences that are just too complex to be enjoyed.

Lastly, it can be argued that, while many western writers tend to want to put the complexity in the flavor, those from the East far more often situate the complexity in the art of making/preparing, which is cognizant that there is only a very limited amount of complexity that can be perceived in the moment. It may also reflect the differing role of food production more generally constraining thinking style (see Henrich, 2014) [102]. In the far east, the tea ceremony, be it the Japanese or Chinese variety, is a carefully choreographed and complex sequence of activities that may take the Master of the Tea Ceremony many years to master (see Anderson, 1991, Okakura, 1989) [103,104]. The complexity is squarely located in the process in the carefully choreographed series of stages that need to be followed in the correct order and much less in the complexity of the tasting experience itself Although beyond the scope of the present manuscript, there are undoubtedly interesting questions here concerning the relationship between notions of complexity as far as the chemical senses are concerned and how complexity (or sensory intricacy) is discussed as far as the other senses are concerned (cf. Snitz, Arzi, Jacobson, Secundo, Weissler, & Yablonka, 2016) [105]. In terms of the design of the menu and the tasting journey, it certainly feels like we have come a long way since one chef had the following to say *“The summit of the chef’s art is to conceive and realize a multicourse meal that progresses through a series of flavors without repetition (S. Hill, The Merchant House, Ludlow, Shropshire).”* (quoted in McGee, 1999) [106]. We have also come a long way from traditional notions that dishes/flavors in a meal should always progress from simple to complex.

Ultimately, then, as this review has hopefully made clear, ‘complexity’ is a term that is used in multiple ways by those working in the world of food and drink. The challenge for the culinary creative given that complexity is so often deemed a desirable attribute of our multisensory tasting experiences, is to communicate that to the consumer in the most effective way since it is not always something that is discernible on the palate itself. This is achieved in a number of ways on both the menu and in the meal itself.

Looking to the future, it will be interesting to determine the extent to which the positive associations that many people have with complexity in food and drink is culture-specific. It will also be interesting to see how in the coming years the desire for complexity interfaces with the growing desire for purity, simplicity, and natural/unadulterated foods (Naessens, 2017) [107]. Given that complexity can be operationalized in ingredients, in preparation, or in the flavor experience. It will also be interesting to see whether the function linking hedonic liking to complexity in the world of food and drink is an inverted U-shape as it has been argued to be the case in the visual arts (Berlyne, 1960, Berlyne et al., 1968) [99,100]. Lastly, in closing, it is worth noting here that early research on the appreciation of visual complexity often described it as a collative property, which means that one’s appreciation of complexity depended on the viewer’s prior experience [108]. The extent to which the same is true in terms of the diner’s or drinker’s perception of complexity in the world of food and drink would also appear to be an interesting question for future research.

## Figures and Tables

**Table 1:** Simple and complex menu item descriptions from McCall & Lynn’s (2008) study [12].

**Filet Mignon**
Low Complexity
10 oz. grilled, mushroom sauce, and served with a choice of potato or vegetable.
High Complexity
10 oz. grilled tenderloin served with a sweet garlic and thyme crust, sliced vine ripe marinated tomato, and smoked mozzarella cheese with a sherry vinegar demi glace.
**Stuffed Breast of Chicken**
Low Complexity
An oven-roasted, stuffed, boneless, skinless chicken breast.Served with wild rice and vegetables.
High Complexity
Cirrus marinated chicken breast stuffed under the skin with shrimp and crabmeat, grilled over a hickory fire, then served with a sweet and spicy Georgia peach sauce, saffron wild rice, and fresh vegetables.
**Pasta**
Low Complexity
Flat egg pasta with smoked chicken, mushromm, a cream sauce, and parmigiano.
High Complexity
Wide flat egg pasta, aauteed with garlic, olive oil, grilled chicken breast, mixed wild mushroom, pancetta(Milan cured bacon), and artichoke hearts in a Pinot Grigio cream sauce, finished with white truffle oil.
